# Histopathological spectrum of ocular masses in a tertiary eye center in Northern China: a 12-year retrospective analysis

**DOI:** 10.3389/fmed.2026.1781259

**Published:** 2026-04-15

**Authors:** Shanshan Ren, Pengfen Wang, Bin Li, Chenming Zhang, Miaomiao Zhang

**Affiliations:** Department of Ophthalmology, Jinan No. 2 People’s Hospital, Jinan, Shandong, China

**Keywords:** eyelid neoplasm, histopathology, ocular mass, orbital lymphoma, retrospective study

## Abstract

**Purpose:**

To characterize the clinicopathological spectrum of ocular masses diagnosed over 12 years at a tertiary referral center in Northern China and identify demographic and regional patterns relevant to clinical practice.

**Methods:**

We retrospectively reviewed 1,345 ocular masses diagnosed at Jinan Second People’s Hospital between February 2014 and July 2025. Cases were identified from pathology archives and included patients with surgical excision or biopsy and definitive histopathology. Data on age, sex, anatomic location, and mass type were analyzed. Classification followed AFIP atlas standards and ICD-O coding.

**Results:**

Of the 1,345 masses, 1,146 (85.2%) were non-malignant and 199 (14.8%) malignant. Lesions were located in the eyelid (35.2%), ocular surface (35.7%), intraocular region (1.8%), and orbit (27.3%). For eyelid masses, nevi (20.8%) and cysts (11.2%) were the most common non-malignant lesions, and basal cell carcinoma (55.6%) and sebaceous gland carcinoma (24.2%) dominated malignancies. For ocular surface masses, the top non-malignant lesions included cysts (20.0%) and inflammatory lesions (18.1%), and malignant melanoma (34.6%) and squamous cell carcinoma (26.9%) were the leading malignancies. Most intraocular masses (20/24) were malignant, and the majority was melanoma (16/20). For orbital masses, hemangiomas (21.1%) were the most frequent benign lesions, and lymphoma (51.9%) was the leading malignancy. The majority of malignancies occurred in patients aged > 60 years (132/199, 66.3%). Compared to patients aged 18–60 years, those aged < 18 years had significantly lower odds of malignancy (OR = 0.21, 95% CI 0.08–0.59), whereas those > 60 years had significantly higher odds of malignancy (OR = 3.29, 95% CI 2.37–4.56).

**Conclusion:**

Most ocular masses were non-malignant. The overall risk of malignancy increased significantly after age 60, and this trend was primarily driven by extraocular lesions.

## 1 Introduction

Ocular masses encompass a heterogeneous group of neoplasms affecting the eyelid, conjunctiva, orbit, and intraocular structures. These lesions range from non-malignant cysts and nevi to sight-threatening and life-threatening malignancies such as uveal melanoma, retinoblastoma, and orbital lymphoma (1). They not only alter facial appearance but also degrade visual function and can be life-threatening. Except for the lens, nearly every ocular tissue can develop space-occupying lesion. If left untreated, malignant ocular tumors may extend locally into the eyelid, orbit, or paranasal sinuses and can invade intracranially or metastasize to distant sites (2).

There has been extensive research on ocular tumors internationally. Currently, WHO EYE5 is the most up-to-date reference of ocular tumor that provides a comprehensive, evidence-based, and multidisciplinary taxonomy (3). Ocular tumors are now classified not only by anatomic site (conjunctiva, eyelid, cornea, uvea, retina, optic nerve, orbit), but also by histologic lineage (epithelial, melanocytic, lymphoid, mesenchymal, neuroectodermal, secondary/metastatic) and increasingly by molecular and biochemical features (4). They are also separated by expected biological behavior, e.g., benign/locally aggressive lesions (e.g., conjunctival papilloma, nevus), premalignant intraepithelial lesions, and malignant tumors with local invasion and metastatic potential (e.g., uveal melanoma, conjunctival squamous cell carcinoma, sebaceous carcinoma). Molecular biology has become an essential and integral part of modern ophthalmic oncology, fundamentally transforming the diagnosis, prognosis, and treatment of eye tumors (5, 6).

Ocular space-occupying lesions are rare, and the incidence of ocular tumors is only about 1 case per 100,000 people (7). However, both the incidence and the prevalence have been increasing over the past decades, driven largely by population aging and improved detection (8). This growing disease burden is not distributed equally, and the epidemiology is shaped by a complex interplay of geography, socioeconomic status, and biological risk factors (9). For example, in many East Asian populations, the incidence of uveal melanoma is lower than in Caucasian populations, while eyelid sebaceous gland carcinoma is relatively more common and is frequently misdiagnosed initially as benign chalazia or chronic blepharitis (10, 11). Sex differences are present in specific categories: orbital lymphoproliferative lesions and orbital metastases commonly mirror sex patterns of their systemic counterparts (e.g., female predominance among breast cancer metastases to the orbit), while the overall sex distribution across all ocular tumors is approximately balanced (12). A bimodal, age-stratified pattern has also been reported: childhood disease is dominated by soft-tissue sarcomas (notably embryonal rhabdomyosarcoma) and other pediatric lesions, whereas adults (especially those aged 50 and older) are predominantly affected by lymphoid and epithelial malignancies, with overall malignancy and metastatic risk rising sharply with age (13, 14). Accurate epidemiological data are essential for guiding clinical diagnosis and prioritizing health resources (15).

While population-based registries (16, 17) provide essential macro-level trends, well-curated hospital-based series (18, 19) are indispensable for capturing granular, real-world clinical context and epidemiologic nuances specific to a local population. In China, Wu et al. (20) reviewed 1,302 eyelid neoplasms treated at Taizhou Hospital and The Third Affiliated Hospital of Wenzhou Medical University from 2013 to 2023 and found that 89% were benign, basal cell carcinoma was the dominant malignancy (74% of malignant cases), malignant cases presented at a substantially older median age (72 vs. 50 years), and sociodemographic index and latitude correlated with the relative proportions of different types of malignancies. Lin et al. (21) analyzed 3,468 ocular surface and orbit tumors treated at Xiamen Eye Center from 2015 to 2020 and found that 91.5% were benign, with malignant lymphoma and basal cell carcinoma being the most common malignancies. Wang et al. (22) conducted a retrospective cohort study of 5,146 biopsy-proven eyelid tumors treated at the Second Affiliated Hospital, Zhejiang University School of Medicine from 2000 to 2018 and noted that 85% were benign (most commonly nevus), basal cell carcinoma was the leading malignancy, sebaceous gland carcinoma comprised a large share of malignancies in older patients, and that malignant tumor composition correlated with sociodemographic index across regions. Yu et al. (23) reviewed 2,228 histologically confirmed eyelid tumors treated at Tianjin Eye Hospital from 2002 to 2015 and found that epidermal (epithelial) tumors were most frequent, the malignant cases (13%) were dominated by basal cell carcinoma and sebaceous gland carcinoma, and malignant lesions occurred at an older mean age and favored the lower eyelid. However, the specific clinicopathological landscape in Shandong remains unknown.

Therefore, this study reviews the clinicopathological features of ocular masses diagnosed at Jinan Second People’s Hospital, a high-volume tertiary hospital in the province of Shandong, to bridge the gap in regional data and inform clinical practice. Our work contributes new value by analyzing a comprehensive, surgically confirmed cohort across all ocular adnexal compartments (eyelid, ocular surface, intraocular, and orbit) within a single tertiary center, thus providing a unified, cross-compartment profile of space-occupying ocular lesions that enables direct comparison of age distribution, anatomical predilection, and malignancy rates across tumor types. While this analysis does not provide population-based incidence, it details the case mix seen in a specialized clinical setting to identify prevalence patterns, particularly regarding malignancy rates in the aging population.

## 2 Materials and methods

### 2.1 Study design and population

We reviewed a retrospective case series of all patients diagnosed with ocular masses at Jinan Second People’s Hospital between February 2014 and July 2025. Cases were identified from the Department of Pathology archives. Inclusion criteria were patients who underwent surgical excision or biopsy with a definitive histopathological diagnosis. Patients with clinical diagnoses unsupported by pathology or incomplete records were excluded.

### 2.2 Data collection and classification

The extracted data included age, sex, anatomical location, and histopathological diagnosis. Anatomical locations were stratified into four categories: eyelid, ocular surface (conjunctiva/cornea), intraocular, and orbit. Tumors were fixed in 4% formaldehyde, embedded in paraffin, sectioned, and stained with hematoxylin and eosin (H&E). Immunohistochemical (IHC) staining was performed when necessary for differential diagnosis (e.g., lymphoid or poorly differentiated tumors). Pathological classification followed the AFIP atlas standards and ICD-O coding (24).

### 2.3 Statistical analysis

Descriptive statistics were used to summarize the data. Age was summarized as median [P25, P75]. Categorical variables were expressed as case count (percentage) and compared across three age groups (< 18 years, 18–60 years, and > 60 years) and four tumor sites using the chi-square test or Fisher’s exact test (with Monte Carlo simulation when applicable) with Holm correction. Odds ratio (ORs) were calculated using the 18–60 years group as the reference. Statistical significance was defined as *P* < 0.05.

## 3 Results

### 3.1 Overview

During the study period, 1,345 ocular tumor cases were diagnosed. The cohort exhibited a nearly balanced sex distribution, comprising 644 (47.9%) males and 701 (52.1%) females. The age distribution was skewed toward adults, with 654 cases (48.6%) in the 18–60 years age group (47 [36, 54] years), 508 cases (37.8%) in the > 60 years group (69 [64, 75] years), and 183 cases (13.6%) in patients aged < 18 years (6 [10, 14] years). The age distribution of the whole cohort was 54 [35, 66] years. Space-occupying lesion were most frequently located on the ocular surface (*n* = 480, 35.7%) and eyelid (*n* = 474, 35.2%), followed by the orbit (*n* = 367, 27.3%) and intraocular structures (*n* = 24, 1.8%). The vast majority were non-malignant (*n* = 1,146, 85.2%) (Table 1).

**TABLE 1:** Clinicopathological Summary of 1,345 ocular masses.

Breakdown^§^	Total (*n* = 1,345, 100%)	Eyelid (*n* = 474, 35.2%)	Ocular Surface (*n* = 480, 35.7%)	Intraocular (*n* = 24, 1.8%)	Orbital (*n* = 367, 27.3%)	*P*
Patient						
Sex^†^		0.014	0.003	0.408	0.446	
Male	644 (47.9%)	205 (43.2%)	256 (53.3%)	14 (58.3%)	169 (46.0%)	0.010
Female	701 (52.1%)	269 (56.8%)	224 (46.7%)	10 (41.7%)	198 (54.0%)	0.010
Age (years)^†^		< 0.001	<0.001	0.779	0.010	
< 18	183 (13.6%)	34 (7.1%)	99 (20.6%)	4 (16.7%)	46 (12.5%)	<0.001
18–60	654 (48.6%)	210 (44.3%)	229 (47.7%)	12 (5.0%)	203 (55.3%)	0.016
> 60	508 (37.8%)	230 (48.5%)	152 (31.7%)	8 (33.3%)	118 (32.2%)	< 0.001
Mass						
Malignance^†^		< 0.001	<0.001	< 0.001	> 0.999	
Non-Malignant	1146 (85.2%)	375 (79.1%)	454 (94.6%)	4 (16.7%)	313 (85.3%)	< 0.001
Malignant	199 (14.8%)	99 (20.9%)	26 (5.4%)	20 (83.3%)	54 (14.7%)	< 0.001

^§^Percentages are calculated based on total cases of the respective category.

† *P*-values of column-wise comparisons are given here.

**FIGURE 1:**
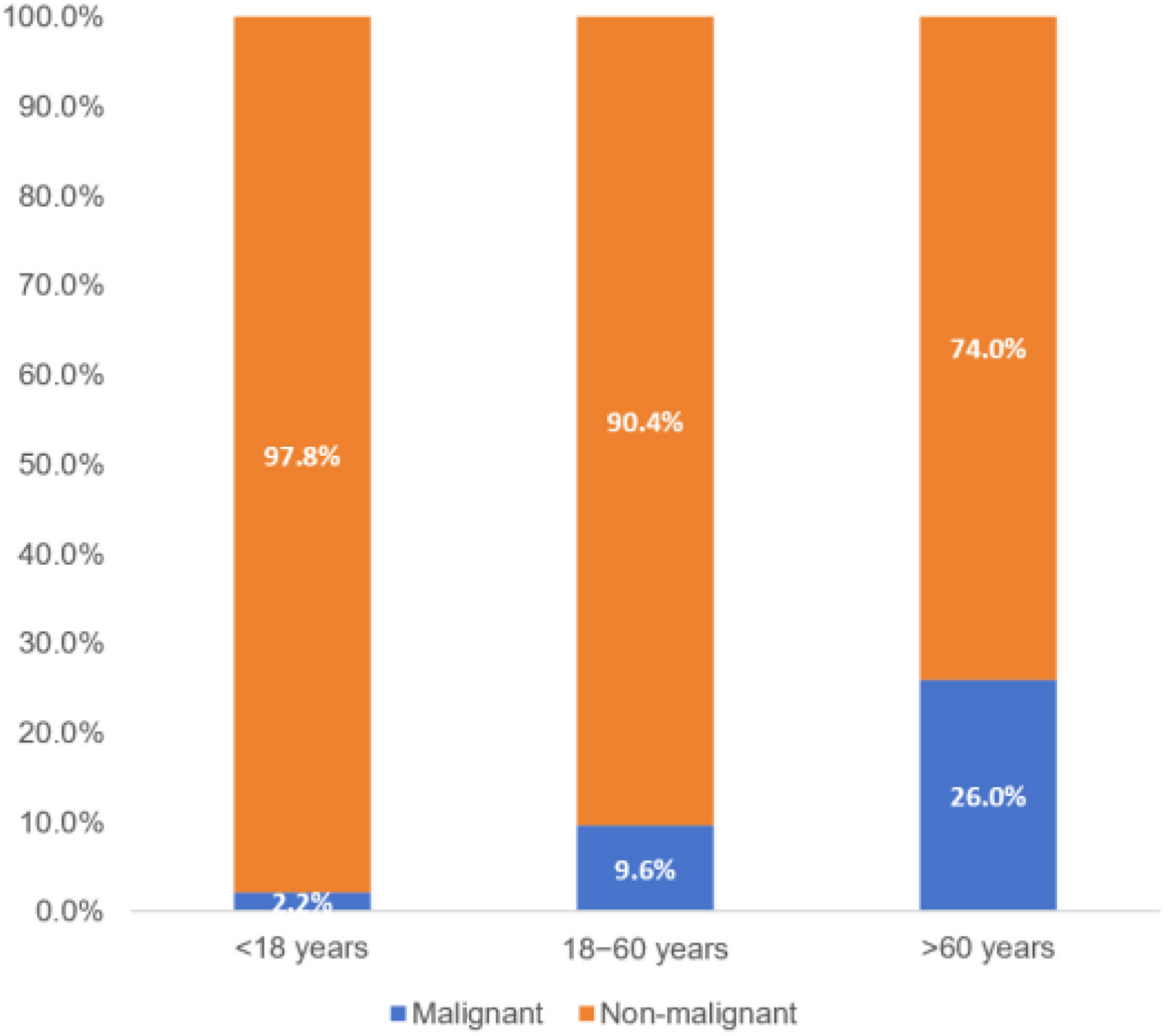
Distribution of malignancy across age groups. Compared to the 18–60 years group, the < 18 years group had a lower odds of malignancy (OR = 0.21, 95% CI 0.08–0.59), whereas the > 60 years group had a higher odds of malignancy (OR = 3.29, 95% CI 2.37–4.56).

**FIGURE 2:**
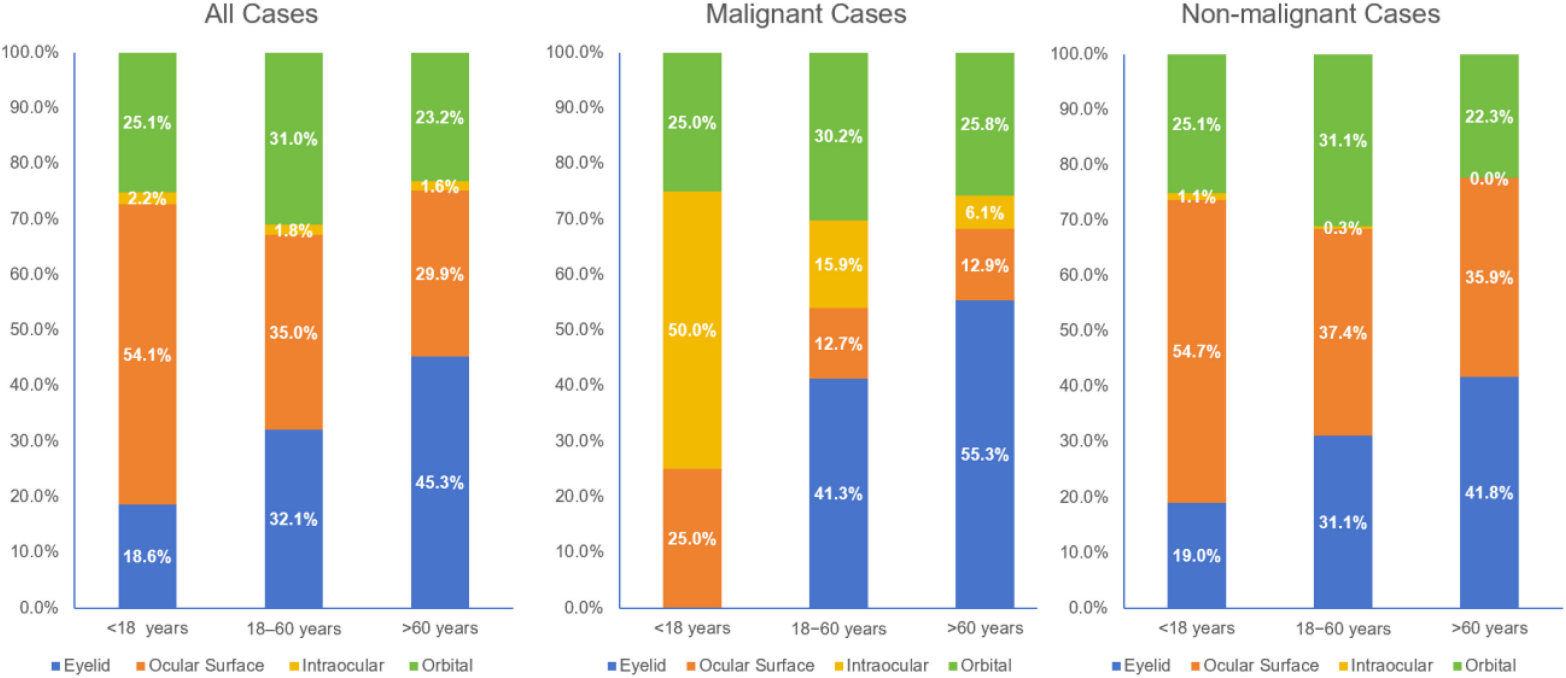
Distribution of ocular mass type across age groups.

Among the 199 (14.8%) malignant cases, 132 cases (66.3%) occurred in patients aged > 60 years, whereas only 4 cases (2.0%) were identified in those aged < 18 years (Table 2). In contrast to the overall cohort, the malignant subset showed a slight male predominance (51.8% vs. 48.2%). Males outnumbered females in all anatomic subtypes except for eyelid malignancies, where females accounted for 51.5% of cases. The distribution of malignancies by location was eyelid (*n* = 99, 49.7%), orbit (*n* = 54, 27.1%), ocular surface (*n* = 26, 13.1%), and intraocular (*n* = 20, 10.1%). Malignancy increased remarkably with age (Figure 1). Compared to patients aged 18–60 years, patients < 18 years had significantly lower odds of malignancy (OR = 0.21, 95% CI 0.08–0.59), whereas patients > 60 years had significantly higher odds (OR = 3.29, 95% CI 2.37–4.56). In addition, the distribution of malignancy was not uniform across anatomical sites (Figure 2 and Table 2). Eyelid malignancies showed the strongest association with the > 60 years age group (73.7%, *P* = 0.019). Conversely, for intraocular malignancies, the 18–60 years age group was dominant (50.0%, *P* = 0.007).

**TABLE 2:** Clinicopathological summary of 199 malignant ocular masses.

Breakdown^§^	Total (*n* = 199, 100%)	Eyelid (*n* = 99, 49.7%)	Ocular surface (*n* = 26, 13.1%)	Intraocular (*n* = 20, 10.1%)	Orbital (*n* = 54, 27.1%)	*P*
Patient						
Sex^†^		0.438	0.986	0.944	0.621	
Male	103 (51.8%)	48 (48.5%)	14 (53.8%)	11 (55.0%)	30 (55.6%)	0.834
Female	96 (48.2%)	51 (51.5%)	12 (46.2%)	9 (45.0%)	24 (44.4%)	0.834
Age (years)^†^		0.019	0.641	0.007	0.833	
< 18	4 (2.0%)	0 (0.0%)	1 (3.8%)	2 (10.0%)	1 (1.8%)	0.016
18–60	63 (31.7%)	26 (26.3%)	8 (30.8%)	10 (50.0%)	19 (35.2%)	0.190
> 60	132 (66.3%)	73 (73.7%)	17 (65.4%)	8 (40.0%)	34 (63.0%)	0.030

^§^Percentages are calculated based on total cases of the respective category.

† *P*-values of column-wise comparisons are given here.

### 3.2 Eyelid masses

The eyelid was a common site of involvement, comprising 474 cases (375 non-malignant, 99 malignant) (Table 3). Among non-malignant lesions, nevi (intradermal, junctional, and compound) were the most prevalent (20.8%), followed by cysts (11.2%) and granulomas (10.9%). Malignancies were dominated by basal cell carcinoma (BCC), which accounted for over half of all eyelid cancers (55.6%). Sebaceous gland carcinoma (SGC) was the second most frequent malignancy (24.2%), and squamous cell carcinoma (SCC) was the third (7.1%).

**TABLE 3:** Histopathological classification of eyelid masses (*n* = 474).

Eyelid mass (*n* = 474)	Case (ratio)^§^
Non-malignant cases (n = 375, 79.1%)	
Nevus	78 (20.8%)
Cyst	42 (11.2%)
Granuloma	41 (10.9%)
Squamous papilloma	36 (9.6%)
Seborrheic keratosis	30 (8.0%)
Hemangioma	20 (5.3%)
Chalazion	12 (3.2%)
Fibroma	9 (2.4%)
Pilomatricoma	9 (2.4%)
Keratoacanthoma	6 (1.6%)
Verruca (wart)	5 (1.3%)
Xanthelasma	4 (1.1%)
Hidradenoma (Sweat gland adenoma)	3 (0.8%)
Others	80 (21.3%)
Subtotal	375 (100.0%)
Malignant Cases (n = 99, 20.9%)	
Basal cell carcinoma	55 (55.6%)
Sebaceous gland carcinoma	24 (24.2%)^¶^
Squamous cell carcinoma	7 (7.1%)
Malignant melanoma	6 (6.1%)
Poorly differentiated carcinoma*	4 (4.0%)
Angiosarcoma	1 (1.0%)
Adenoid cystic carcinoma	1 (1.0%)
MALT lymphoma^†^	1 (1.0%)
Subtotal	99 (100.0%)

^§^Percentages are calculated based on total cases of the respective category.

† Extranodal marginal zone B-cell lymphoma of mucosa-associated lymphoid tissue. ^¶^20 cases were confirmed as meibomian gland carcinoma.

* Carcinoma that lacks distinctive histological features and cannot be classified into conventional subtypes.

### 3.3 Ocular surface masses

Ocular surface masses (conjunctiva and cornea) totaled 480 cases (Table 4), the majority of which were non-malignant (*n* = 454, 94.6%). Cysts (20.0%), inflammatory lesions (18.1%), and nevi (16.3%) were the most common diagnoses. Malignancies of the ocular surface were rare (*n* = 26, 5.4%). Among them, malignant melanoma was the most frequent (34.6%), followed closely by squamous cell carcinoma (26.9%) and squamous cell carcinoma *in situ* (19.2%).

**TABLE 4:** Histopathological classification of ocular surface masses (*n* = 480).

Ocular surface masses (*n* = 480)	Case (ratio)^§^
Non-malignant cases (n = 454, 94.6%)	
Cyst	91 (20.0%)
Inflammatory lesions	82 (18.1%)
Nevi	74 (16.3%)
Dermoid cyst	41 (9.0%)
Squamous papillomas	36 (7.9%)
Lipomas	24 (5.3%)
Hemangioma	18 (4.0%)
Polyp	14 (3.1%)
Epibulbar choristoma	7 (1.5%)
Myxoma	5 (1.1%)
Keratoacanthoma	4 (0.9%)
Others	58 (12.8%)
Subtotal	454 (100.0%)
Malignant cases (n = 26, 5.4%)	
Malignant melanoma	9 (34.6%)
Squamous cell carcinoma	7 (26.9%)
Squamous cell carcinoma *in situ*	5 (19.2%)
Lymphoma	2 (7.7%)
Basal cell carcinoma	2 (7.7%)
Meibomian gland carcinoma	1 (3.8%)
Subtotal	26 (100.0%)

^§^Percentages are calculated based on total cases of the respective category.

### 3.4 Intraocular masses

Intraocular masses were the least common category (*n* = 24, 1.8%) but they accounted for the highest proportion of malignant masses (83.3%). The malignancies (*n* = 20) included uveal malignant melanomas (16/20, 80.0%), retinoblastoma (2/20, 10.0%), and squamous cell carcinoma (2/20, 10.0%). Non-malignant lesions were rare and included inflammatory lesions and cysts (Table 5).

**TABLE 5:** Histopathological classification of intraocular masses (*n* = 24).

Intraocular mass (*n* = 24)	Case (ratio)^§^
Non-malignant cases (n = 4, 16.7%)	
Inflammatory lesions	2 (50.0%)
Dermoid cyst	1 (25.0%)
Iris cyst	1 (25.0%)
Subtotal	4 (100.0%)
Malignant cases (n = 20, 83.3%)	
Malignant melanoma	16 (80.0%)
Retinoblastoma	2 (10.0%)
Squamous cell carcinoma	2 (10.0%)
Subtotal	20 (100.0%)

^§^Percentages are calculated based on total cases of the respective category.

### 3.5 Orbital masses

Orbital lesions comprised 367 cases (313 benign, 54 malignant). Non-malignant lesions were most frequently hemangiomas (21.1%), which was followed by cysts (16.9%) and idiopathic orbital inflammatory pseudotumors (11.8%). Among the 54 orbital malignancies, lymphoma was the predominant type (51.9%), the majority being B-cell origin. Other major malignancies included adenocarcinoma/adenoid cystic carcinoma (14.8%) and malignant melanoma (7.4%) (Table 6).

**TABLE 6:** Histopathological classification of orbital masses (*n* = 367).

Orbital mass (*n* = 367, 27.3%)	Case (ratio)^§^
Non-malignant cases (n = 313, 85.3%)	
Hemangioma	66 (21.1%)
Cyst	53 (16.9%)
Idiopathic orbital inflammatory pseudotumor	37 (11.8%)
Pleomorphic adenoma	27 (8.6%)
Lymphoproliferative lesion	24 (7.7%)
Lipoma	17 (5.4%)
Fibrous histiocytoma	13 (4.2%)
Neurofibroma	6 (1.9%)
Squamous papilloma	4 (1.3%)
Pilomatricoma	4 (1.3%)
Schwannomas	3 (1.0%)
Teratomas	3 (1.0%)
IgG4-related dacryoadenitis/sialadenitis^†^	2 (0.6%)
Hamartoma	2 (0.6%)
Osteoma	2 (0.6%)
Other	50 (16.0%)
Subtotal	313 (100.0%)
Malignant cases (n = 54, 14.7%)	
Lymphoma	28 (51.9%)
Adenocarcinoma and adenoid cystic carcinoma	8 (14.8%)
Malignant melanoma	4 (7.4%)
Meibomian gland carcinoma	3 (5.6%)
Basal cell carcinoma	3 (5.6%)
Squamous cell carcinoma	2 (3.7%)
Small cell undifferentiated sarcoma	2 (3.7%)
Small round blue cell tumor	1 (1.9%)
Poorly differentiated angiosarcoma	1 (1.9%)
Liposarcoma	1 (1.9%)
Follicular carcinoma	1 (1.9%)
Subtotal	54 (100.0%)

^§^Percentages are calculated based on total cases of the respective category.

† Mikulicz’s disease.

## 4 Discussion

This study presents a comprehensive histopathological analysis of 1,345 ocular masses (including ocular tumors) managed at a major tertiary referral center in Northern China over a 12-year period. Our findings define a distinct clinicopathological profile characterized by a predominance of non-malignant lesions (85.2%), and 66.3% of the malignant tumors occurred in patients over 60 years of age. While these results reflect the specific case mix of a specialized institution, they serve as a valuable sentinel for disease patterns in the populous Shandong region, particularly when contrasted with data from Southern China and Western cohorts.

### 4.1 Eyelid malignancy: validation of the Asian pattern

The distribution of eyelid malignancies in our cohort strongly demonstrates the uniqueness of the Asian population in epidemiology. While BCC remained the most common malignancy (55.6%), consistent with global literature, the proportion of SGC was disproportionately high, accounting for 24.2% of all eyelid cancers. This figure aligns closely with other Chinese studies, such as Wang et al. in South China (34.24%) (22) and Yu et al. in Tianjin (34.6%) (23), but stands in stark contrast to findings from Thagaard et al. and Pe’er et al. where SGC typically comprises less than 5% of eyelid neoplasms (25, 26).

The high burden of SGC in this Northern Chinese population is clinically significant. SGC is notoriously aggressive and often mimics benign inflammatory conditions like chalazia or blepharitis, leading to diagnostic delays (27, 28). The consistently high proportion of SGC across both Northern and Southern Chinese cohorts suggests a stable ethnic or genetic predisposition rather than a purely latitude-dependent environmental factor. Consequently, ophthalmologists in the East Asian region must maintain a low threshold for biopsy in elderly patients presenting with recurrent or atypical eyelid nodules.

### 4.2 Ocular surface and environmental factors

Ocular surface masses were predominantly non-malignant space-occupying lesions (94.6%), with cysts, inflammatory lesions, and nevi being the majority. Among the rare malignancies (*n* = 26), malignant melanoma (34.6%) SCC (26.9%), and SCC *in situ* (19.2%) were the primary entities. The relatively low absolute number of ocular surface SCCs compared to tropical regions likely reflects the temperate latitude of the Shandong Province, as cumulative ultraviolet (UV) radiation is the primary environmental driver for ocular surface squamous neoplasia (OSSN) (29, 30). Lifestyle changes that reduce outdoor activity may have also contributed to the low rate of malignancy. However, the presence of actinic-related lesions underscores the necessity for public health education regarding ocular UV protection, even in non-tropical zones (31).

### 4.3 Intraocular masses: surgical selection and epidemiological context

Intraocular masses constituted the smallest anatomical subgroup, representing only 1.8% (24/1,345) of all cases. This low frequency is characteristic of histopathology-based series and must be interpreted through the lens of clinical management. Unlike eyelid or conjunctival lesions, which are readily accessible for excisional biopsy, intraocular masses are often diagnosed via non-invasive multimodal imaging (e.g., B-scan ultrasonography, OCT, angiography). Non-malignant intraocular lesions, such as choroidal nevi or hemangiomas, are typically managed via observation rather than surgery. Consequently, the pathology archives inherently select for lesions requiring enucleation or resection, resulting in a disproportionately high malignancy rate (32, 33).

In our series, 83.3% (20/24) of intraocular masses were malignant, the highest ratio among all anatomical sites, and the most common malignancy was malignant melanoma (16/20, 80.0%). Choroidal melanoma (CM) is the most common primary intraocular malignant tumor in adults. It is highly aggressive; because its clinical manifestations are diverse and sometimes atypical, it is easily misdiagnosed as choroidal hemorrhage, subretinal hemorrhage, or retinal pigment epithelium hemorrhage with organization and scarring. However, in view of the population of our catchment area, the finding of only two retinoblastoma cases over 12 years contradicts expected incidence rates. Retinoblastoma occurs in approximately 1 in 15,000–20,000 live births (34). Given the population density of the Shandong province, a single-center volume of < 0.2 cases per year strongly suggests a referral filter. Parents may bypass provincial hospitals in favor of national centers in Beijing or Shanghai to access advanced globe-salvaging therapies (e.g., intra-arterial chemotherapy). Our data may have accurately captured the adult burden of uveal melanoma, which is treated locally via enucleation or plaque brachytherapy, but is unlikely to have captured the pediatric burden, as complex pediatric cases may be migrated to top-tier metropolises.

Whereas eyelid malignancies were dominated by the > 60 years age group (73.7%, *P* = 0.019), the peak incidence of intraocular malignancies was found for the 18–60 years age group (50.0%, *P* = 0.007). This distinct epidemiological pattern aligns with the existing literature. For example, Shields et al. (35). found that uveal melanomas are most commonly present in the adult age group of 21–60 years. The current finding emphasizes that regarding intraocular malignancy, clinicians should maintain a high level of suspicion across all adult age groups, rather than relying on advanced age as a primary risk indicator.

### 4.4 Orbital pathology and the rise of lymphoma

In the orbit, our findings mirror the global trend of increasing lymphoma incidence. Malignant lymphoma constituted 51.9% of all orbital malignancies, the vast majority being of B-cell origin. This aligns with recent meta-analyses indicating that orbital lymphoma rates are rising worldwide, with Asian populations showing a particular predilection for the MALT (Mucosa-Associated Lymphoid Tissue) subtype (36, 37). Age is a key factor in these lymphoma cases, as 22 of the 28 patients were > 60 years old. In contrast, non-malignant cases were dominated by vascular lesions (hemangiomas, 21.1%), which is consistent with the classic distributions reported in regional and international series (38, 39).

### 4.5 Limitations

Several limitations characterize this study. First, as a retrospective, single-center series, our data are subject to selection bias and do not represent population-based prevalence. Second, the exclusion of clinically diagnosed benign lesions (e.g., small nevi managed observationally) skews our benign-to-malignant ratio toward surgically managed cases. Finally, the aforementioned referral patterns may have caused underrepresentation of pediatric intraocular malignancies.

## 5 Conclusion

In conclusion, this 12-year analysis confirms that while most ocular masses were non-malignant, the proportion of malignant masses was markedly higher in patients over 60 years of age, a trend primarily driven by extraocular lesions. Age-based risk assessment must be interpreted in the context of tumor location, as intraocular malignancies do not follow the same age-associated pattern seen in extraocular disease. The current findings provide a robust baseline for regional clinical practice and future development of multi-center registry.

## Data Availability

The original contributions presented in the study are included in the article/supplementary material, further inquiries can be directed to the corresponding author.
